# Prolonged Grief Disorder: Psychometric Validation of Criteria Proposed for *DSM-V* and *ICD-11*

**DOI:** 10.1371/journal.pmed.1000121

**Published:** 2009-08-04

**Authors:** Holly G. Prigerson, Mardi J. Horowitz, Selby C. Jacobs, Colin M. Parkes, Mihaela Aslan, Karl Goodkin, Beverley Raphael, Samuel J. Marwit, Camille Wortman, Robert A. Neimeyer, George Bonanno, Susan D. Block, David Kissane, Paul Boelen, Andreas Maercker, Brett T. Litz, Jeffrey G. Johnson, Michael B. First, Paul K. Maciejewski

**Affiliations:** 1 Department of Psychiatry, Brigham and Women's Hospital, Boston, Massachusetts, United States of America; 2 Center for Psycho-Oncology and Palliative Care Research, Dana Farber Cancer Institute, Boston, Massachusetts, United States of America; 3 Harvard Medical School Center for Palliative Care, Boston, Massachusetts, United States of America; 4 Department of Psychiatry, University of California School of Medicine, San Francisco, California, United States of America; 5 Department of Psychiatry, Yale University School of Medicine, New Haven, Connecticut, United States of America; 6 St. Christopher's Hospice, Sydenham, and St. Joseph's Hospice, Hackney, England; 7 Department of Internal Medicine, Yale University School of Medicine, New Haven, Connecticut, United States of America; 8 Department of Psychiatry and Behavioral Neurosciences, Cedars-Sinai Medical Center, Los Angeles, California, United States of America; 9 Department of Psychiatry and Biobehavioral Sciences, David Geffen School of Medicine, University of California at Los Angeles, Los Angeles, California, United States of America; 10 Department of Population Mental Health and Disasters, University of Western Sydney Medical School, New South Wales, Australia; 11 Department of Psychology, University of Missouri, St. Louis, Missouri, United States of America; 12 Department of Psychology, State University of New York at Stony Brook, New York, United States of America; 13 Department of Psychology, The University of Memphis, Memphis, Tennessee, United States of America; 14 Department of Counseling and Clinical Psychology, Teachers College, Columbia University, New York, New York, United States of America; 15 Department of Psychiatry and Behavioral Sciences, Memorial Sloan-Kettering Cancer Center, New York, New York, United States of America; 16 Department of Clinical and Health Psychology, Utrecht University, Utrecht, The Netherlands; 17 Department of Clinical Psychology, University of Zürich, Zürich, Switzerland; 18 Veterans Affairs Boston Healthcare System, Boston, Massachusetts, United States of America; 19 National Center for PTSD, Boston, Massachusetts, United States of America; 20 Boston University School of Medicine, Boston, Massachusetts, United States of America; 21 Department of Psychiatry, Columbia University, New York, New York, United States of America; University of Cambridge, United Kingdom

## Abstract

**Background:**

Bereavement is a universal experience, and its association with excess morbidity and mortality is well established. Nevertheless, grief becomes a serious health concern for a relative few. For such individuals, intense grief persists, is distressing and disabling, and may meet criteria as a distinct mental disorder. At present, grief is not recognized as a mental disorder in the *DSM-IV* or *ICD-10*. The goal of this study was to determine the psychometric validity of criteria for prolonged grief disorder (PGD) to enhance the detection and potential treatment of bereaved individuals at heightened risk of persistent distress and dysfunction.

**Methods and Findings:**

A total of 291 bereaved respondents were interviewed three times, grouped as 0–6, 6–12, and 12–24 mo post-loss. Item response theory (IRT) analyses derived the most informative, unbiased PGD symptoms. Combinatoric analyses identified the most sensitive and specific PGD algorithm that was then tested to evaluate its psychometric validity. Criteria require reactions to a significant loss that involve the experience of yearning (e.g., physical or emotional suffering as a result of the desired, but unfulfilled, reunion with the deceased) and at least five of the following nine symptoms experienced at least daily or to a disabling degree: feeling emotionally numb, stunned, or that life is meaningless; experiencing mistrust; bitterness over the loss; difficulty accepting the loss; identity confusion; avoidance of the reality of the loss; or difficulty moving on with life. Symptoms must be present at sufficiently high levels at least six mo from the death and be associated with functional impairment.

**Conclusions:**

The criteria set for PGD appear able to identify bereaved persons at heightened risk for enduring distress and dysfunction. The results support the psychometric validity of the criteria for PGD that we propose for inclusion in *DSM-V* and *ICD-11*.

*Please see later in the article for Editors' Summary*

## Abstract 2

Holly Prigerson and colleagues tested the psychometric validity of criteria for prolonged grief disorder (PGD) to enhance the detection and care of bereaved individuals at heightened risk of persistent distress and dysfunction.

## Editors' Summary

**Background:**

Virtually everyone loses someone they love during their lifetime. Grief is an unavoidable and normal reaction to this loss. After the death of a loved one, bereaved people may feel sadness, anger, guilt, anxiety, and despair. They may think constantly about the deceased person and about the events that led up to the person's death. They often have physical reactions to their loss—problems sleeping, for example—and they may become ill. Socially, they may find it difficult to return to work or to see friends and family. For most people, these painful emotions and thoughts gradually diminish, usually within 6 months or so of the death. But for a few people, the normal grief reaction lingers and becomes increasingly debilitating. Experts call this complicated grief or prolonged grief disorder (PGD). Characteristically, people with PGD have intrusive thoughts and images of the deceased person and a painful yearning for his or her presence. They may also deny their loss, feel desperately lonely and adrift, and want to die themselves.

**Why Was This Study Done?:**

PGD is not currently recognized as a mental disorder although it meets the requirements for one given in the American Psychiatric Association's *Diagnostic and Statistical Manual of Mental Disorders*, *4^th^ Edition (DSM-IV)* and in the World Health Organization's *International Statistical Classification of Diseases and Related Health Problems*, *10^th^Edition (ICD-10)*. Before PGD can be recognized as a mental disorder (and included in *DSM-V* and *ICD-11*), bereavement and mental-health experts need to agree on standardized criteria for PGD. Such criteria would be useful because they would allow researchers and clinicians to identify risk factors for PGD and to find ways to prevent PGD. They would also help to ensure that people with PGD get appropriate treatments such as psychotherapy to help them change their way of thinking about their loss and re-engage with the world. Recently, a panel of experts agreed on a consensus list of symptoms for PGD. In this study, the researchers undertake a field trial to develop and evaluate algorithms (sets of rules) for diagnosing PGD based on these symptoms.

**What Did the Researchers Do and Find?:**

The researchers used “item response theory” (IRT) to derive the most informative PGD symptoms from structured interviews of nearly 300 people who had recently lost a close family member. These interviews contained questions about the consensus list of symptoms; each participant was interviewed two or three times during the two years after their spouse's death. The researchers then used “combinatoric” analysis to identify the most sensitive and specific algorithm for the diagnosis of PGD. This algorithm specifies that a bereaved person with PGD must experience yearning (physical or emotional suffering because of an unfulfilled desire for reunion with the deceased) and at least five of nine additional symptoms. These symptoms (which include emotional numbness, feeling that life is meaningless, and avoidance of the reality of the loss) must persist for at least 6 months after the bereavement and must be associated with functional impairment. Finally, the researchers show that individuals given a diagnosis of PGD 6–12 months after a death have a higher subsequent risk of mental health and functional impairment than people not diagnosed with PGD.

**What Do These Findings Mean?:**

These findings validate a set of symptoms and a diagnostic algorithm for PGD. Because most of the study participants were elderly women who had lost their husband, further validation is needed to check that these symptoms and algorithm also apply to other types of bereaved people such as individuals who have lost a child. For now, though, these findings support the inclusion of PGD in *DSM-V* and *ICD-11* as a recognized mental disorder. Furthermore, the availability of a standardized way to diagnose PGD will help clinicians identify the minority of people who fail to adjust successfully to the loss of a loved one. Hopefully, by identifying these people and helping them to avoid the onset of PGD (perhaps by providing psychotherapy soon after a death) and/or providing better treatment for PGD, it should now be possible to reduce the considerable personal and societal costs associated with prolonged grief.

**Additional Information:**

Please access these Web sites via the online version of this summary at http://dx.doi.org/10.1371/journal.pmed.1000121.

- This study is further discussed in a *PLoS Medicine* Perspective by Stephen Workman
- The Dana Farber Cancer Institute has a page describing its Center for Psycho-oncology and Palliative Care Research
- The UK Royal College of Psychiatrists has a leaflet on bereavement (in English, Welsh, Urdu, and Chinese)
- The US National Cancer Institute also has information about coping with bereavement for patients and health professionals (in English and Spanish)
- MedlinePlus has links to other information about bereavement (in English and Spanish)
- The *Journal of the American Medical Association* has a patient page on abnormal grief
- Harvard Medical School provides a short family health guide about complicated grief
- Information on DSM-IV and ICD-10 is available

## Introduction

Bereavement is a universal experience to which most individuals adequately adjust. Nevertheless, numerous studies have shown that bereaved individuals have higher rates of disability and medication use than their nonbereaved counterparts [1]–[7], and are themselves at heightened risk of death [8]–[11]. The excess morbidity and mortality is likely to be concentrated in a grief-stricken few. The challenge has been to identify vulnerable bereaved individuals so that interventions could reduce their risk of adverse outcomes.

Following a major loss, such as the death of a spouse, a noteworthy minority of bereaved individuals experiences “a clinically significant behavioral or psychological syndrome or pattern that occurs in an individual and that is associated with present distress or disability” [12]. These are the requirements for meeting the definition of a mental disorder in the *Diagnostic Statistical Manual of Mental Disorders*, 4th Edition (*DSM-IV*) [12]. Nevertheless, the *DSM-IV* excludes grief as a disorder on the grounds that it is “an expectable and culturally sanctioned response to a particular event” [12]. In the *DSM-IV*, bereavement is classified as a “V” code; that is, an “other condition that may be a focus of clinical attention” [12]. Similarly, in the *International Statistical Classification of Diseases and Related Health Problems, Tenth Revision* (*ICD-10*) [13], bereavement is classified as a “Z” code, which refers to “occasions when circumstances other than a disease or injury result in an encounter or are recorded by providers as problems or factors that influence care” [14].

The *DSM-IV and ICD-10* focus on the distinction between “normal” grief and major depressive disorder (MDD), but neglect to acknowledge that grief, per se, may be pathological. Studies have shown that symptoms denoting complicated, or prolonged, grief are distinguishable from symptoms of uncomplicated grief [15]–[18] and that only the former are associated with significant impairment [15]–[25]. The aim of the present study is to validate criteria for prolonged grief disorder (PGD) proposed for inclusion in *DSM-V* and *ICD-11*. The justification for validating criteria for PGD and proposing inclusion in *DSM-V* and *ICD-11* lies in the distinctive phenomenology, etiology, course, response to treatment, and adverse outcomes associated with PGD symptoms.

PGD symptomatology—variously referred to as “complicated grief” (CG) [15],[17]–[20],[22],[25]–[28], “traumatic grief” (TG) [21],[23],[24],[29], and complicated grief disorder (CGD) [22]—have repeatedly been shown to be different from the symptoms of other *DSM-IV* and *ICD-10* disorders (e.g., MDD). For example, in studies of bereaved individuals from a variety of different countries, yearning loads highly on the grief factor, but not on depression or anxiety factors, whereas sadness loads highly only on a depression factor, and feeling nervous and worried loads highly only on an anxiety factor [15],[19],[20],[26],[28]–[31]. A study of negative cognitions among bereaved individuals found that being overwhelmed by the loss (i.e., “If I would fully realize what the death of ___ meant, I would go crazy”) was a cognition specific to PGD, but not depression [32]. The distinction between the symptoms of grief and depressive symptoms found in bereaved individuals has also been shown in advanced cancer patients [30] and caregivers of nursing home residents with advanced dementia [31],[33].

The set of risk factors and clinical correlates of PGD includes a history of childhood separation anxiety [34], controlling parents [35], parental abuse or death [36], a close kinship relationship to the deceased (e.g., parents) [37],[38], insecure attachment styles [39], marital supportiveness and dependency [39],[40], and lack of preparation for the death [41],[42]—all suggesting that attachment issues are salient in creating a vulnerability to PGD. For example, we find that feelings of emotional dependency on the dying patient is associated with symptoms of grief, but not with symptoms of depression in patient caregivers [39] and recently bereaved persons [40]. We have also found that childhood separation anxiety uniquely predicts PGD, but not MDD, posttraumatic stress disorder (PTSD), or generalized anxiety disorder (GAD) following bereavement later in life [34]. The identified grief symptoms have been shown not to relate to the changes of electroencephalographic (EEG) sleep physiology associated with MDD [43]. Most recently, a functional magnetic resonance imaging (fMRI) study by O'Connor et al. [44] has demonstrated that only patients with complicated grief showed reward-related neural activity in the nucleus accumbens in response to reminders of the deceased. The nucleus accumbens cluster “was positively associated with yearning, but not with time since death, participant age, or positive/negative affect” [44]. Taken together, these findings suggest distinct clinical correlates of grief symptoms relative to depressive symptoms.

PGD symptoms also demonstrate incremental validity in that they are associated with elevated rates of suicidal ideation and attempts, cancer, immunological dysfunction, hypertension, cardiac events, functional impairments, hospitalization, adverse health behaviors, and reduced quality of life in adults [19],[21],[24],[25],[30],[45] and in children and adolescents [46], after controlling for the effects of depression and/or anxiety. In a Swedish sample of bereaved parents 4–9 y after the death of their child from cancer, parents with unresolved grief were at risk for long-term mental and physical impairments, increased health service use, and increased sick leave over and above the effects of depression and anxiety [47]. These findings highlight the enduring nature of bereavement-related distress and disability, and the societal consequences of unresolved grief.

The course and response to treatment of PGD differ from those of normal grief [48],[49] and depression [48]–[52]. Tricyclic antidepressants alone and with interpersonal psychotherapy have proven ineffective relative to placebo for the reduction of PGD symptoms [50]–[52]. By contrast, randomized, controlled trials of psychotherapy designed specifically for PGD have demonstrated efficacy for PGD symptom reduction [53],[54]. The efficacy of a PGD-specific treatment highlights the benefits of an accurate diagnosis.

Although the results above suggest that symptoms of grief constitute a syndrome that operationally defines a mental disorder, no agreed upon and tested diagnostic algorithm for PGD exists. Psychiatrists such as Lindemann [55], Parkes [56], Raphael [57], Horowitz [22], and Jacobs [58] have noted the suffering associated with intense and/or chronic mourning. Nevertheless, no explicit criteria developed from a consensus process have been assessed and then tested in a community-based sample of bereaved individuals. The absence of explicit and agreed upon (i.e., standardized) criteria for PGD has hampered efforts for its inclusion in the *DSM* and *ICD* systems. The establishment of standardized criteria for PGD would enable researchers to investigate the prevalence, risk factors, outcomes, neurobiology, prevention, and treatment of this disorder. Such criteria would also assist clinicians in the accurate detection and treatment, as well as reimbursement for treatment, of PGD.

As a first step toward the development of consensus criteria for PGD, we convened a group of experts in bereavement, mood and anxiety disorders, and psychiatric nosology to review the evidence justifying the development of diagnostic criteria [23]. Following the panel's conclusion that the evidence merited the development of a diagnostic algorithm for a grief disorder, they engaged in a 2-d workshop culminating in the formulation of consensus criteria. A preliminary testing of the criteria analyzed the most relevant, yet incomplete, data available at the time—data lacking the full set of proposed consensus criteria and an independent rating to diagnose PGD. Results of this preliminary report supported the sensitivity and specificity of the initially proposed algorithm [23].

Here, we report the results of a field trial designed specifically to develop and evaluate diagnostic algorithms for PGD based on symptoms proposed by the consensus panel. The aim of this study was to establish the psychometric validity of, and propose criteria for, a new syndrome, PGD.

## Methods

### Sample

Data were obtained for the Yale Bereavement Study (YBS) (e.g., [25],[34]–[36],[40],[41],[45],[48]), a National Institute of Mental Health (NIMH)-funded (MH56529) investigation to conduct a field trial of consensus criteria [23] for PGD. The YBS was a longitudinal, interview-based study of community-dwelling bereaved individuals in Connecticut. The YBS was approved by the institutional review boards (IRBs) of all participating sites and was in accordance with Health Insurance Portability and Accountability Act (HIPAA) regulations.

Recruitment involved locating family survivors bereaved 6 mo or less found on contact lists of the Greater Bridgeport/Fairfield American Association of Retired Persons (AARP) Widowed Persons Service (WPS), a community-based outreach program. The contact lists provided names of recently widowed persons who a volunteer widowed person would contact to describe the WPS program. Fewer than 5% of those contacted participated in any WPS program; the lists included those approached, but not necessarily actively involved in, the WPS. A comparison between vital records and the WPS contact list revealed that WPS listings provide an unbiased and comprehensive ascertainment of recently widowed people. Participants were also recruited from pastoral care offices in the New Haven area. Participants from this alternative source (117/317 = 37.0% of study participants) did not differ significantly from WPS participants (200/317 = 63.0% of study participants) on gender, income, education, race/ethnicity, or quality of life, but they were younger than WPS participants (*p* = 0.05) (mean age = 59.7 y, standard deviation [SD] = 16.4 versus 63.2 y, SD = 11.5 y, respectively).

Of the 575 potential participants contacted, 317 (55.1%) agreed to participate. Reasons for nonparticipation included reluctance to participate in research (*n* = 11; 4.3%); being too busy (*n* = 46; 17.8%) or too upset (*n* = 27; 10.5%); “doing fine” (*n* = 23; 8.9%); “not interested” or “no reason” (*n* = 145; 56.2%); and “other” reasons (*n* = 6; 2.3%). Nonparticipants were more likely to be male (37.2% versus 25.9%, *p*<0.001) and older (mean age = 68.8 y versus 61.7 y, *p*<0.001) than participants. Written informed consent was obtained from all study participants. Interviews were conducted by master's degree–level interviewers trained by YBS investigators (HGP, SCJ). Interviewers were required to demonstrate nearly perfect agreement (κ≥0.90) with the YBS investigators for diagnoses of psychiatric disorders (e.g., MDD) and PGD in five pilot interviews before being permitted to interview for the study.

The 317 YBS participants were interviewed at baseline an average of 6.3 mo (SD = 7.0 mo) post-loss. First follow-up interviews (*n* = 296, 93.4% of participants) were completed an average of 10.9 mo (SD = 6.1 mo) post-loss; second follow-up interviews (*n* = 263, 83.0% of participants) at an average of 19.7 mo (SD = 5.8 mo) post-loss. The average age of participants was 61.8 y (SD = 18.7 y), most were female (73.7%), white (95.3%), educated beyond high school (60.4%), and spouses of the deceased (83.9%). Data were restructured such that assessments were grouped into more uniform post-loss time periods (0–6 mo, 6–12 mo, and 12–24 mo post-loss). A single assessment within each time period was randomly selected for each participant to remove cases in which two assessments occurred within the same interval. The present study sample (*n* = 291; 91.8% of YBS participants) included participants interviewed at least once within 12 mo post-loss and who provided sufficient information to evaluate PGD.

### Assessment of Symptoms of PGD

Symptoms of PGD were assessed with the rater version of the Inventory of Complicated Grief—Revised (ICG-R) [34]–[36],[40],[41],[45],[59], a structured interview designed to assess a wide variety of potential PGD symptoms, using five-point scales to represent increasing levels of symptom severity. The ICG-R is a modification of the Inventory of Complicated Grief (ICG) [15] that includes all the symptoms proposed by the consensus panel [23] and additional symptoms enabling the testing of alternative diagnostic algorithms [22]. The ICG-R and the original ICG have both proven highly reliable (e.g., [15],[25],[36],[41]) (e.g., Cronbach's α>0.90; test-retest reliability coefficient = 0.80 [15]) and to possess criterion validity [15],[21],[24],[25],[45]. Based on prior work [23],[59], a symptom was considered present if rated 4 or 5, and absent if rated 1, 2, or 3, on its five-point scale. Interviewers were trained by project investigators (HGP, SJC) to provide a separate evaluation of whether or not the participant represented a current “case” of PGD.

### Assessment of Psychiatric Disorders

Psychiatric disorders were assessed using the Structured Clinical Interview for *DSM-IV* (SCID Non-Patient Version) [60]. The rater-administered SCID assessed criteria for *DSM-IV* anxiety disorders (GAD, PTSD, and panic disorder [PD]) and mood disorders (MDD). Research has supported the reliability and validity of SCID diagnoses [61].

### Assessment of Additional Outcomes

Positive responses to one or more of the four Yale Evaluation of Suicidality [25] screening questions were categorized as having suicidal ideation. The Established Populations for Epidemiological Studies of the Elderly [62] measured performance of activities of daily living [63] and physical functioning [64]. Individuals with at least “some difficulty” with at least one of the 14 tasks (e.g., bathing) were considered functionally impaired in order to make the measure sensitive to impairment in a highly functioning sample. Scores less than 5 (below the lowest quartile) on the Medical Outcomes Short-Form [65] indicated inferior quality of life.

### Analyses and Results

The psychometric validation of diagnostic criteria for PGD proceeded through a cumulative series of analyses, with each phase in the overall analysis having a distinct aim. In Phase 1 of the analysis, the aim was to limit the set of candidate symptoms for PGD to those that were informative and unbiased. In Phase 2 of the analysis, the goal was to construct an objective, reliable, valid symptom criterion standard for PGD by which to evaluate alternative diagnostic algorithms for meeting symptom criteria for PGD. The aim of Phase 3 of the analysis was to identify a specific, optimum diagnostic algorithm for meeting symptom criteria for PGD among a large set of candidate algorithms. Phase 4 of the analysis was designed to evaluate the predictive validity for temporal subtypes of meeting the optimal symptom criteria for PGD as an empirical means to inform the specification of a “timing criterion” for the diagnosis of PGD. In Phase 5, the goal was to propose a complete set of “*DSM*-style” diagnostic criteria based on results of the preceding phases in the analysis. Phase 6 of the analysis evaluated the predictive validity of the final, complete, proposed criteria for PGD.

#### Phase 1: deriving a set of informative, unbiased symptoms of PGD

**Figure 1:**
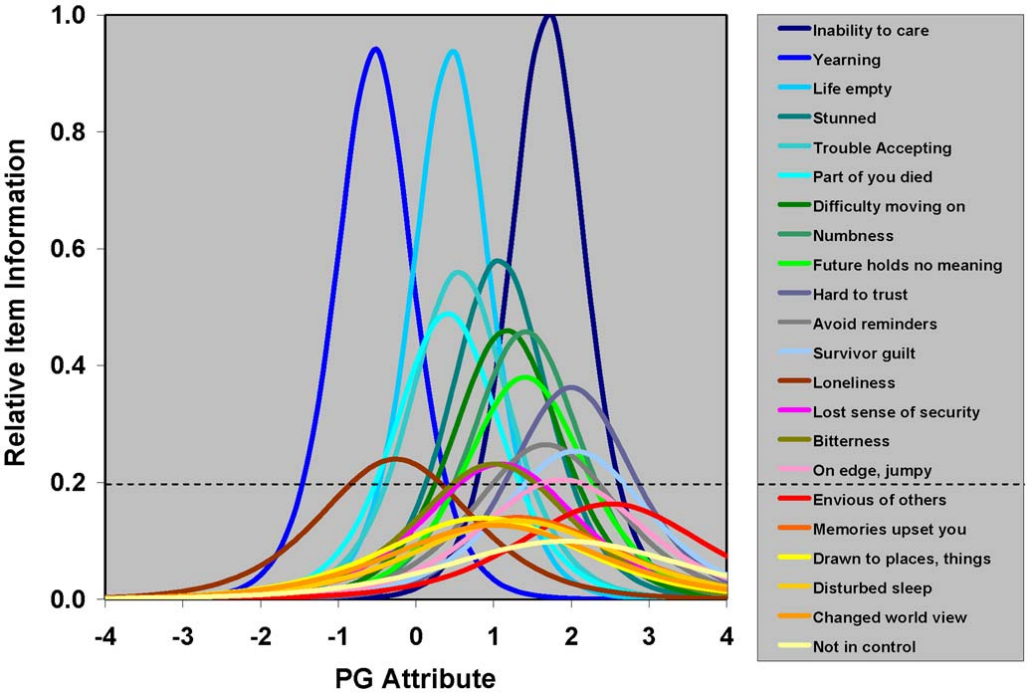
Relative item information as a function of the prolonged grief attribute for 22 candidate symptoms for PGD. IRT IIF analysis of 22 binary candidate symptoms for PGD was performed using a 2-PL IRM. This figure displays item information as a function of the PG attribute for all 22 of these symptoms included in this IRM, relative to the maximum information for the most informative symptom, “inability to care about others since the death.” The horizontal line in the figure represents the standard used to discriminate between 16 informative candidate symptoms retained for further analysis, and six uninformative candidate symptoms excluded from further analysis (as indicated in Table 1).

**Figure 2:**
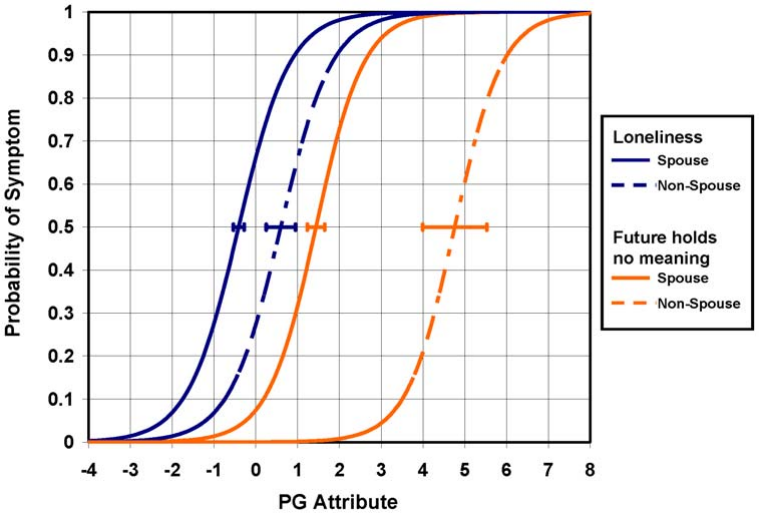
Differential item functioning for two biased symptoms. IRT DIF analysis of candidate symptoms for PGD was performed with respect to age (less than 65 y versus greater than or equal to 65 y), gender (male versus female), education (beyond versus not beyond high school), relationship to the deceased (spouse versus nonspouse), and time from loss (0–6 mo versus 6–12 mo post-loss). This figure displays IRT item characteristic curves (ICCs) for two symptoms found to differ with respect to relationship to the deceased (spouse versus nonspouse). The horizontal error bar associated with each ICC represents the standard error in the estimate of the location of the ICC with respect to the PG attribute. Of 16 informative symptoms examined, four symptoms displayed DIF and were excluded from further analysis (as indicated in Table 1).

IRT [66], item information function (IIF) analysis, and differential item functioning (DIF) analysis were used to evaluate candidate symptoms for PGD assessed 0–12 mo post-loss. IIF analysis was used to evaluate the amount of information about the prolonged grief (PG) “attribute” (underlying construct) provided by each of 22 dichotomous candidate symptoms for PGD. Consistent with the use of IRT to construct a one-dimensional scale for PG, Cattell's scree test [67] indicated that grief, as measured by these 22 symptoms, is one-dimensional. Figure 1 presents item information functions for these 22 symptoms derived from a two-parameter logistic (2-PL) item response model (IRM). Within the framework of IRT, information for a given value of the latent PG attribute is inversely related to its conditional standard error of measurement. Greater information implies lower measurement error, and greater measurement precision, for PG. Six symptoms with maximum “peak” information less than 20% of that of the most informative symptom were considered to be relatively uninformative and removed from further consideration as possible symptoms for assessing and diagnosing PGD. DIF analysis of between-group differences in item location parameters was used to evaluate potential biases in the assessment of the remaining 16 informative, candidate symptoms for PGD with respect to age (less than 65 y versus greater than or equal to 65 y), gender (male versus female), education (beyond versus not beyond high school), relationship to the deceased (spouse versus nonspouse), and time from loss (0–6 versus 6–12 mo post-loss). Figure 2 displays item characteristic curves by group, spouse, and nonspouse, for two items eliminated from consideration as possible symptoms for assessing and diagnosing PGD due to evidence of DIF using a 16-item 2-PL IRM. A total of four of 16 informative symptoms were found to be biased with respect to time from loss, gender, and/or relationship to the deceased. Table 1 provides a summary of results for these IRT IIF and DIF analyses of candidate symptoms for assessing and diagnosing PGD.

**Table 1:** Evaluation of candidate symptoms for PGD (*n* = 287).

Candidate PGD Symptom	Rate (%)	IRT IIF Analysis^a^		IRT DIF Analysis^b^		
		*I*_max_ c	Θ_max_ d	Sex	Spouse	Time
**Inability to care about others since the death**	6.6	1.00	1.70		**Biased**	
Yearning for, or preoccupation with, deceased	68.3	0.94	−0.53			
Life empty, meaningless without deceased	34.8	0.93	0.46			
Stunned, dazed, or shocked about the death	19.2	0.58	1.07			
Trouble accepting the death	32.7	0.56	0.56			
Feel part of you died along with the deceased	37.6	0.49	0.41			
Difficulty moving on with life without deceased	18.1	0.46	1.17			
Sense of numbness since the death	13.6	0.46	1.41			
**Future holds no meaning without the deceased**	14.6	0.38	1.40		**Biased**	
Hard for you to trust others since the death	7.0	0.36	2.00			
Avoid reminders of deceased	12.5	0.26	1.67			
Survivor guilt	8.4	0.25	2.04			
**Loneliness as a result of the death**	57.1	0.24	−0.26	**Biased**	**Biased**	**Biased**
**Lost sense of security since the death**	23.3	0.23	1.09	**Biased**		
Bitterness or anger related to the death	25.1	0.23	1.01			
On edge, jumpy since the death	11.5	0.20	1.88			
**Envious of others who have not lost someone close**	7.0	**0.16**	2.51			
**Memories of the deceased upset you**	22.6	**0.14**	1.31			
**Drawn to places, things associated with deceased**	31.0	**0.14**	0.86			
**Disturbed sleep since the death**	23.3	**0.13**	1.28			
**The death has shattered your world view**	28.6	**0.13**	1.02			
**Lost sense of control since the death**	16.4	**0.10**	1.93			

Relatively uninformative (*I*_max_<0.20) or biased symptoms are displayed in **bold font**. All others are informative, unbiased symptoms and were retained for further analysis.

a IRT IIF analysis was performed using all 22 symptoms, showing 16 to be informative (*I*_max_≥0.20).

b IRT DIF analysis was restricted to 16 relatively informative symptoms, showing four to be biased.

c *I*_max_ represents maximum item information relative to that of “inability to care about others….”

d Θ_max_ represents location of *I*_max_ for the item along the PG attribute scale.

The following 12 informative, unbiased ICG-R symptoms were retained for consideration in a diagnostic algorithm: yearning; avoidance of reminders of the deceased; disbelief or trouble accepting the death; a perception that life is empty or meaningless without the deceased; bitterness or anger; emotional numbness or detachment from others; feeling stunned, dazed or shocked; feeling part of oneself died along with the deceased; difficulty trusting others; difficulty moving on with life; on edge or jumpy; survivor guilt (Cronbach's α = 0.82).

#### Phase 2: deriving a criterion standard for assessing diagnostic algorithms for symptoms of PGD

In the absence of an established, standard method for diagnosing PGD, there was a need to develop a criterion standard for “caseness” of PGD by which the performance of alternative algorithms for PGD could be evaluated. As a potential criterion standard for PGD, the rater determination of caseness of PGD had the advantage of reflecting experienced clinical judgment. However, rater assessments of PGD were subjective, were made without explicit reference to any established criteria, and were not always consistent with more objective, reliable assessments of PG as measured with IRM scores for the underlying PG attribute (i.e., raters assign PGD to some individuals with low scores, and not to others with high scores, on the PG attribute scale). It was decided that a criterion standard for caseness should be informed by clinical judgment, but should also be a function of PG symptom severity. Dichotomized IRM PG attribute scores, informed by rater assessments of PGD, would provide objective, reliable, valid criterion standard diagnoses for PGD.

**Figure 3:**
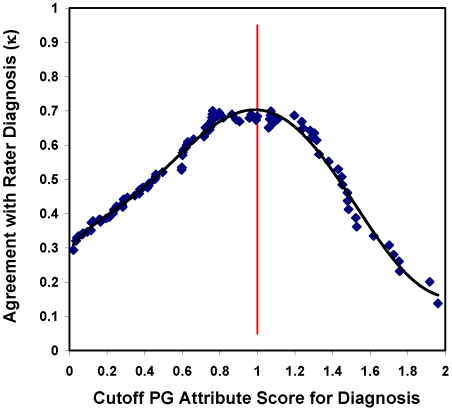
Agreement between rater diagnoses and dichotomized prolonged grief attribute score diagnoses of PGD as a function of cutoff PG attribute score for diagnosis. Dichotomized IRM PG attribute scores provide objective, reliable criterion standard diagnoses for PGD. This figure illustrates how rater diagnoses were used to establish a minimum-threshold cutoff PG attribute score for diagnosis of PGD (i.e., PG attribute score≥minimum-threshold cutoff PG attribute score). An optimal cutoff PG attribute score of 1 maximized agreement between rater diagnoses and dichotomized IRM PG attribute score diagnoses of PGD.

Scores from a 2-PL IRM for PG based on the 12 informative, unbiased symptoms were used to order individuals in terms of PG symptom severity. Agreement between rater and minimum-threshold PG attribute assessments of caseness of PGD was maximized to establish an optimum minimum threshold for caseness of PGD along this IRM scale. As illustrated in Figure 3, PGD “cases” determined by a minimum-threshold “cutoff” PG attribute score of approximately 1.0 had the greatest agreement (κ = 0.68) with cases determined by the rater assessments. An IRM PG attribute score above this minimum-threshold cutoff value became our criterion standard for PGD caseness.

#### Phase 3: identifying an optimal diagnostic algorithm for symptoms of PGD

Based on consensus opinion of the previously mentioned expert panel [23], and confirmed by results showing yearning was the most common (68.3%) and most informative (*I*_max_ = 0.94) of the 12 items and provided the maximum information and the lowest degree of severity (Θ_max_ = −0.53), yearning was specified as a mandatory symptom. The analyses then sought to determine the number and combination of the remaining 11 (nonmandatory) symptoms in addition to yearning that would yield the most efficient (i.e., optimum balance between sensitive and specific) diagnosis for PGD with respect to our criterion standard. Combinatorics [68], the branch of mathematics that studies the number of different ways of arranging sets, was used to enumerate alternative sets of nonmandatory symptoms to construct alternative, candidate diagnostic algorithms for meeting the symptom criterion for PGD. Each of these diagnostic algorithms was specified in terms one common, mandatory symptom, yearning, a specific set of *n* other, nonmandatory symptoms, and some minimum number of nonmandatory symptoms within this set, *k*, which one must have to satisfy the symptom criterion for PGD. A total of 4,785 of these algorithms for meeting the symptom criterion for PGD were enumerated [i.e., the sum of (11 choose *n*×(*n*−3) for *n* = 5, 6, 7, 8, 9, where (11 choose *n*) represents the number of ways of choosing *n* of 11 nonmandatory symptoms, *n* ∈ {5, 6, 7, 8, 9}, and (*n*−3) represents the number of values of *k* ∈ {3, …, *n*−1}, considered for a given value of *n*] and subsequently evaluated with respect to the criterion standard. Algorithms requiring yearning and as few as three of five, and as many as eight of nine, additional symptoms were considered.

**Figure 4:**
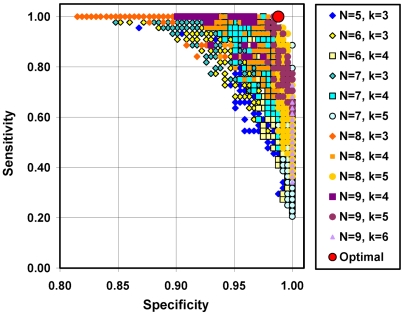
Alternative diagnostic algorithms for meeting symptom criteria for PGD. Each data point in this figure represents the performance, in terms of sensitivity and specificity with respect to a criterion standard for PGD, of a unique “*DSM*-style” diagnostic algorithm for meeting symptom criteria for PGD. Each algorithm is specified in terms of one common, mandatory symptom, yearning, a specific set of *n* other, nonmandatory symptoms, and some minimum number of nonmandatory symptoms within this set, *k*, which one must have to satisfy the symptom criterion for PGD. Based on the current data, the optimal, most efficient algorithm requires having yearning and at least five of the following nine symptoms: avoidance of reminders of the deceased; trouble accepting the death; a perception that life is empty or meaningless without the deceased; bitterness or anger related to the loss; emotional numbness; feeling stunned, dazed or shocked; feeling that part of oneself died along with the deceased; difficulty in trusting others; and difficulty moving on with life.

Figure 4 displays results for a subset of the diagnostic algorithms considered. Each data point in Figure 4 represents the sensitivity and specificity of a unique diagnostic algorithm. The optimal, most efficient algorithm included yearning and at least five of the nine following symptoms: avoidance of reminders of the deceased; disbelief or trouble accepting the death; a perception that life is empty or meaningless without the deceased; bitterness or anger related to the loss; emotional numbness; feeling stunned, dazed, or shocked; feeling part of oneself had died along with the deceased; difficulty trusting others; difficulty moving on with life (sensitivity = 1.00; specificity = 0.99; positive predictive value = 0.94; negative predictive value = 1.00). The optimal algorithm displayed convergent validity with respect to the previously proposed diagnostic algorithm for PGD (κ = 0.68) and the rater diagnosis of PGD (κ = 0.68), and discriminant validity with respect to other mood and anxiety disorders (Φwith MDD = 0.36; PTSD = 0.31; GAD = 0.17).

#### Phase 4: evaluating the predictive validity for temporal subtypes of PGD

Three subtypes of PGD were defined in terms of patterns of meeting diagnostic criteria for PGD at 0–6 and 6–12 mo post-loss: acute = meeting the symptom criteria for PGD at 0–6 mo, but not at 6–12 mo, post-loss; delayed = meeting the symptom criteria for PGD at 6–12 mo, but not at 0–6 mo, post-loss; and persistent = meeting the symptom criteria for PGD both at 0–6 and at 6–12 mo post-loss. In Table 2, we see that the acute temporal specification was not significantly associated with any of the examined outcomes evaluated 12–24 mo post-loss. Delayed was significantly (*p*<0.001) associated with suicidal ideation and poor quality of life. Persistent was significantly (*p*<0.01) associated with mental disorders (MDD, PTSD, or GAD), suicidal ideation, and poor quality of life. Delayed or persistent was significantly (*p*<0.05) associated with psychiatric disorders (MDD, PTSD, or GAD), suicidal ideation, functional disability, and poor quality of life. These results indicate that diagnoses of PGD before 6 mo post-loss do *not* effectively identify bereaved individuals at risk of long-term dysfunction, whereas delayed and persistent temporal subtypes do.

**Table 2:** Mental health and functional consequences of meeting symptom criteria for PGD by temporal subtype.

Outcome (12–24 Mo Post-Loss)	Relative Risk for Outcome Associated with PGD Temporal Subtype:							
	Acute (15/172 [8.7%])^a^		Delayed (6/172 [3.5%])^a^		Persistent (12/172 [7.0%])^a^		Delayed or persistent (28/242 [11.6%])^b^	
	RR	95% CI	RR	95% CI	RR	95% CI	RR	95% CI
MDD, PTSD, or GAD	1.54	(0.20–11.98)	3.86	(0.55–27.22)	11.58***	(4.41–30.43)	10.19***	(4.72–21.99)
Suicidal ideation (*n* = 171:241)^c^	1.97	(0.64–6.09)	4.93***	(1.92–12.64)	3.29*	(1.28–8.43)	4.44***	(2.62–7.53)
Functional disability (*n* = 170:240)^c^	0.51	(0.18–1.45)	1.54	(0.73–3.25)	1.40	(0.79–2.50)	1.65**	(1.16–2.34)
Poor quality of life (*n* = 168:238)^c^	0.76	(0.20–2.89)	3.78***	(1.93–7.40)	2.58*	(1.23–5.41)	3.17***	(2.03–4.95)

Acute = meeting symptom criteria at 0–6 mo, but not at 6–12 mo, post-loss; Delayed = not meeting symptom criteria at 0–6 mo, but meeting symptom criteria at 6–12 mo post-loss; Persistent = meeting symptom criteria at 0–6 and 6–12 mo post-loss.

a The denominator included those assessed at both 0–6 and 6–12 mo post-loss.

b The denominator included all those assessed at 6–12 mo post-loss, regardless of the 0–6-mo post-loss assessment.

c Sample sizes (*n*) varied due to missing data. The first number in the parenthesis represents *n* for those assessed at both 0–6 and 6–12 mo post-loss, the second number after the colon represents *n* for those assessed at 6–12 mo post-loss regardless of the 0–6-mo post-loss assessment.

*p<0.05; **p<0.01; ***p<0.001.

CI, confidence interval; RR, relative risk.

#### Phase 5: proposing criteria for PGD

To reduce further the likelihood of a false-positive diagnosis, a timing criterion (Criterion D) was added to specify that a diagnosis not be made until at least 6 mo have elapsed since the death. This would exclude the acute cases described above in which a person with initially high levels of grief in the first few months experiences declines in grief intensity at and beyond 6 mo post-loss. To be conservative in our diagnosis of PGD, we also added a requirement that the symptomatic distress be associated with functional impairment (Criterion E).

The ultimate consensus criteria set for PGD proposed for *DSM-V* and *ICD-11* appears in Table 3. Diagnoses of PDG based on these criteria demonstrated convergent validity with respect to the diagnostic algorithm proposed by Horowitz et al. [22] (κ = 0.69) and the rater diagnosis of PGD (κ = 0.52), and discriminant validity with respect to other mood and anxiety disorders (Φ with MDD = 0.48; PTSD = 0.23; GAD = 0.21).

**Table 3:** Criteria for PGD proposed for *DSM-V* and *ICD-11*.

Category	Definition
A.	Event: Bereavement (loss of a significant other)
B.	Separation distress: The bereaved person experiences yearning (e.g., craving, pining, or longing for the deceased; physical or emotional suffering as a result of the desired, but unfulfilled, reunion with the deceased) daily or to a disabling degree.
C.	Cognitive, emotional, and behavioral symptoms: The bereaved person must have five (or more) of the following symptoms experienced daily or to a disabling degree:
	1. Confusion about one’s role in life or diminished sense of self (i.e., feeling that a part of oneself has died)
	2. Difficulty accepting the loss
	3. Avoidance of reminders of the reality of the loss
	4. Inability to trust others since the loss
	5. Bitterness or anger related to the loss
	6. Difficulty moving on with life (e.g., making new friends, pursuing interests)
	7. Numbness (absence of emotion) since the loss
	8. Feeling that life is unfulfilling, empty, or meaningless since the loss
	9. Feeling stunned, dazed or shocked by the loss
D.	Timing: Diagnosis should not be made until at least six months have elapsed since the death.
E.	Impairment: The disturbance causes clinically significant impairment in social, occupational, or other important areas of functioning (e.g., domestic responsibilities).
F.	Relation to other mental disorders: The disturbance is not better accounted for by major depressive disorder, generalized anxiety disorder, or posttraumatic stress disorder.

#### Phase 6: evaluating the predictive validity of the proposed criteria for PGD

Among those not concurrently meeting *DSM* criteria for MDD, PTSD, or GAD (*n* = 215), PGD diagnoses assessed 6–12 mo post-loss (7/215 = 3.3%) were significantly (*p*<0.01) associated with psychiatric diagnoses (MDD, PTSD, or GAD), suicidal ideation, functional disability, and low quality of life 12–24 mo post-loss (see Table 4). Among those concurrently meeting *DSM* criteria for MDD, PTSD, or GAD (*n* = 27), PGD diagnoses 6–12 mo post-loss (10/27 = 37.0%) were significantly associated with psychiatric diagnoses (MDD, PTSD, or GAD) at 12–24 mo post-loss (relative risk = 2.38, *p* = 0.043).

**Table 4:** Mental health and functional impairment at 12–24 mo post-loss associated with PGD among those not meeting *DSM* criteria for MDD, PTSD, or GAD at 6–12 mo post-loss (*n* = 215).

Outcome (12–24 Mo Post-Loss)	PGD Diagnosis (6–12 Mo Post-Loss)			
	Yes (3.3%)	No (96.7%)	RR	95% CI
MDD, PTSD, or GAD	28.6%	3.4%	8.49**	(2.14–33.72)
Suicidal ideation^a^ (*n* = 214)	57.1%	10.1%	5.63***	(2.64–12.03)
Functional disability^a^ (*n* = 213)	71.4%	35.9%	1.99**	(1.20–3.29)
Poor quality of life^a^ (*n* = 210)	83.3%	14.7%	5.67***	(3.48–9.22)

a Sample sizes (*n*) varied due to missing data.

*p<0.05; **p<0.01; ***p<0.001.

CI, confidence interval; RR, relative risk.

## Discussion

Our results indicate that PGD meets *DSM* criteria for inclusion as a distinct mental disorder on the grounds that it is a clinically significant form of psychological distress associated with substantial disability. Findings from this field trial of consensus criteria for PGD confirm prior work demonstrating the distinctiveness of the symptoms of PGD (e.g., [15],[18]–[20],[22],[26],[27],[29]–[31]). The proposed diagnostic algorithm for PGD has quite incomplete overlap with established mental disorders commonly occurring among recently bereaved individuals (MDD, PTSD). Further, our results indicate that in the absence of mental disorders found in *DSM-IV* (e.g., MDD), the proposed algorithm for PGD predicts substantial dysfunction—impairment missed by the current psychiatric diagnostic system. Because standard treatments for depression have not always proven effective for the reduction of PGD [49]–[52], whereas psychotherapies designed specifically to ameliorate symptoms of PGD have demonstrated efficacy [53],[54], there exists a need for the accurate detection and specialized treatment of PGD.

Although the YBS data may appear unrepresentative of the general US population, a comparison with US Census 2005 [48],[69],[70] data reveals similarities with the US widowed population. For example, the YBS sample was 73.7% female compared with 80.7% of the US widowed population and 95.3% white compared with 80.2% of the US widowed population. Like the population of US widowed individuals, the YBS sample is disproportionately female, white, and elderly. Compared with the US widowed population, however, the study participants were somewhat younger, more likely to be male, and a higher proportion was white and better educated. Future research should replicate the analyses in older, nonwhite, less-educated widowed samples.

Although there is a need to confirm the results in nonwidowed bereaved persons, we consider widowhood following an older spouse's death from natural causes to be the prototypical case of bereavement. In the US, 84% of all deaths occur among individuals who are 65 y and over [71], and less than 7% of deaths are from unnatural causes (e.g., unintentional injuries, assault, suicide) [72]. Given that in later life one's spouse/partner is the person most likely to be adversely affected by the death, a sample of older widowed persons surviving the death of a spouse from natural causes provides an important sample in which to develop and test criteria for a bereavement-related mental disorder. In addition, the symptoms retained were only those proven to be invariant across gender, time from loss, and kinship groups (e.g., IRT DIF analysis removed items that performed differently based on whether or not the deceased was a spouse) and a distinct advantage of IRT is that it produces generalizable results regardless of sample characteristics [66]. Thus, the results are expected to be generalizable to most bereaved individuals. The generalizability of the results reported here is not intended to deny the value in further confirmation of the findings in nonwidowed, more traumatically bereaved, younger, less-educated, more male, and ethnically and geographically diverse samples, and the need to examine longer-term bereavement outcomes (e.g., 3, 5, and 10 y post-loss).

Although the sample size may appear modest, the study was designed and appropriately powered to evaluate a wide range of potential diagnostic criteria (i.e., the first phases of the analyses used the full sample [*n* = 291]). The YBS PGD prevalence rate was obtained in a resilient community sample in which rates of mental illness were lower than those that have been reported in other bereavement studies (e.g., 9% for MDD compared with 22% in the first year of widowhood) [73]. The only analyses limited by statistical power would have been the predictive validity analyses. Here, we found large, statistically significant effects suggesting the conservative nature of our estimates of functional impairment associated with PGD.

Study participants may have been less distressed than study nonparticipants. Given the relatively low rates of MDD in the YBS sample and that 10.5% refused participation in the YBS due to being “too upset,” the prevalence rate of PGD reported here may be an underestimate. In addition, our statistical power to detect significant effects of PGD on mental health and functional impairment outcomes would be lower than would have been the case if more distressed nonparticipants with PGD had been included in the study sample.

### Conclusion

This report provides psychometric validation of a diagnostic algorithm for PGD. Although further validation work will, no doubt, be needed, we consider the evidence sufficient to justify PGD's serious consideration for inclusion in *DSM-V* and *ICD-11*. In light of the recent concerns about financial conflicts of interest in psychiatric research, especially that which involves pharmaceutical manufacturers, it is noteworthy that this study was federally funded by the US NIMH, and no part of this research was sponsored by producers of a potential therapeutic remedy for PGD.

Although most bereaved individuals will eventually adapt to the loss of a significant other more or less successfully, a significant, identifiable minority will experience chronic and disabling grief. A PGD diagnosis has the potential to enhance the detection and effective treatment of a substantial cause of morbidity among persons who have experienced the loss of a significant other. The diagnosis and treatment of PGD offers the promise of reducing the personal and societal toll taken by prolonged grief.

## Abbreviations

2-PL: two-parameter logistic
DIF: differential item functioning
*DSM-IV*: *Diagnostic Statistical Manual of Mental Disorders*, 4th Edition
GAD: generalized anxiety disorder
*ICD-10*: *International Statistical Classification of Diseases and Related Health Problems*
ICG-R: Inventory of Complicated Grief—Revised
IIF: item information function
IRM: item response model
IRT: item response theory
MDD: major depressive disorder
PG: prolonged grief
PGD: prolonged grief disorder
PTSD: posttraumatic stress disorder
SCID: Structured Clinical Interview for *DSM-IV*
WPS: Widowed Persons Service
YBS: Yale Bereavement Study
