# Correction: Prolonged Grief Disorder: Psychometric Validation of Criteria Proposed for *DSM-V* and *ICD-11*

**DOI:** 10.1371/annotation/a1d91e0d-981f-4674-926c-0fbd2463b5ea

**Published:** 2013-12-17

**Authors:** Holly G. Prigerson, Mardi J. Horowitz, Selby C. Jacobs, Colin M. Parkes, Mihaela Aslan, Karl Goodkin, Beverley Raphael, Samuel J. Marwit, Camille Wortman, Robert A. Neimeyer, George A. Bonanno, Susan D. Block, David Kissane, Paul Boelen, Andreas Maercker, Brett T. Litz, Jeffrey G. Johnson, Michael B. First, Paul K. Maciejewski

Author 'George Bonanno' should be listed as 'George A. Bonanno'.

